# Supplementary material for: Lack of strong evidence for cone photoreceptors’ contribution to human melatonin suppression and alerting response to light
Source: iScience. 2025 Jun 23;28(7):112983. doi: 10.1016/j.isci.2025.112983 (PMC12273515; doi:10.1016/j.isci.2025.112983)
Supplement: Document S1. Figures S1–S7 and Tables S1–S7 [file mmc1.pdf]

iScience, Volume *28*

Supplemental information

Lack of strong evidence for cone photoreceptors' contribution to human melatonin suppression and alerting response to light

Fatemeh Fazlali, Rafael Lazar, Faady Yahya, Christian Epple, Manuel Spitschan, Oliver Stefani, and Christian Cajochen

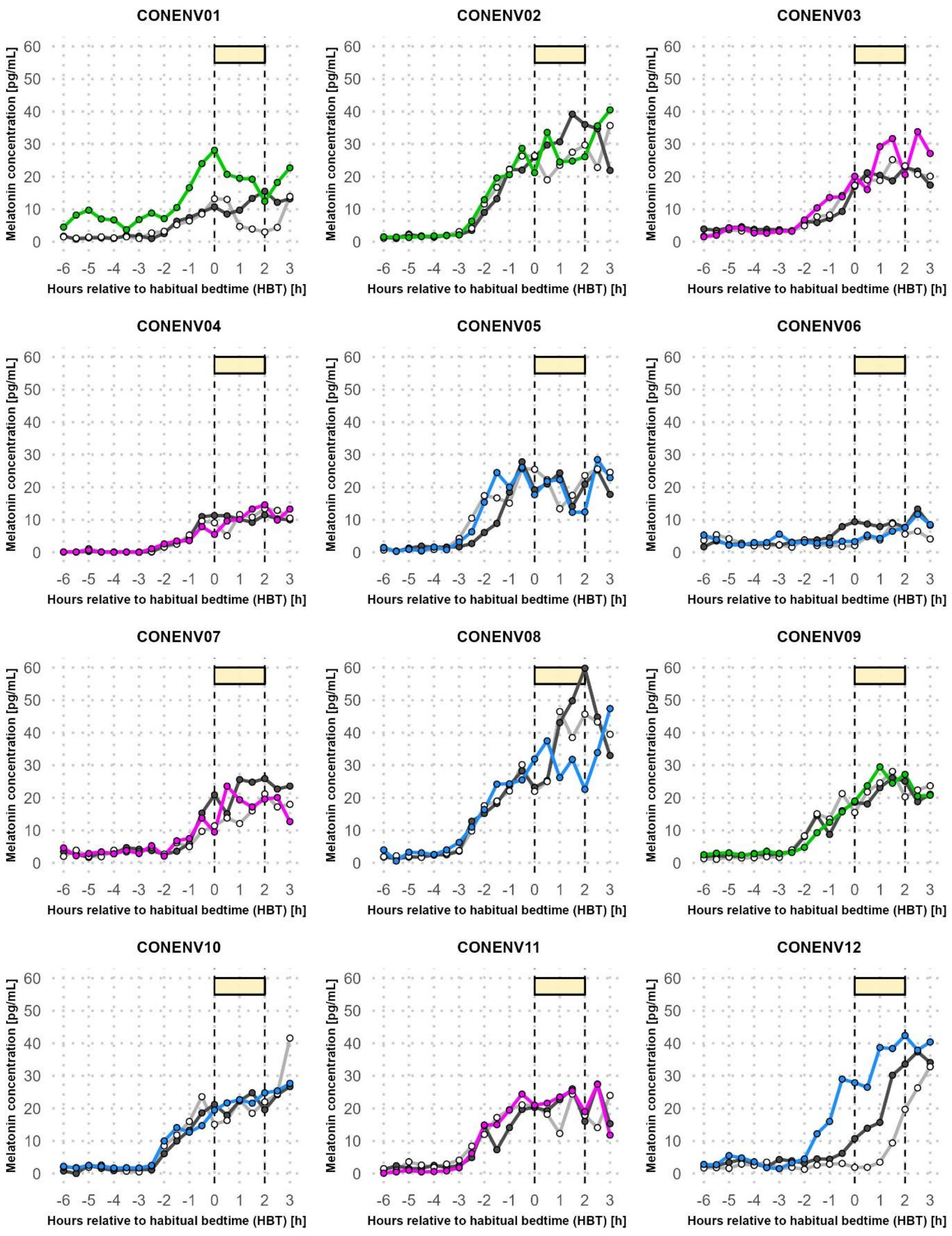

Figure S1. Individual salivary melatonin concentration profiles under each of the three light conditions for participants 1-12. Each panel represents the melatonin concentration (pg/ml) of a participant over time, relative to their habitual bedtime (HBT). Data are presented for the three experimental light conditions: Baseline Light (BL; black), Background Light (BG; gray) and Cone-Modulated Flickering Light (S, M-L, S+M+L; blue, green, pink). The dashed vertical lines indicate the duration of light exposure.

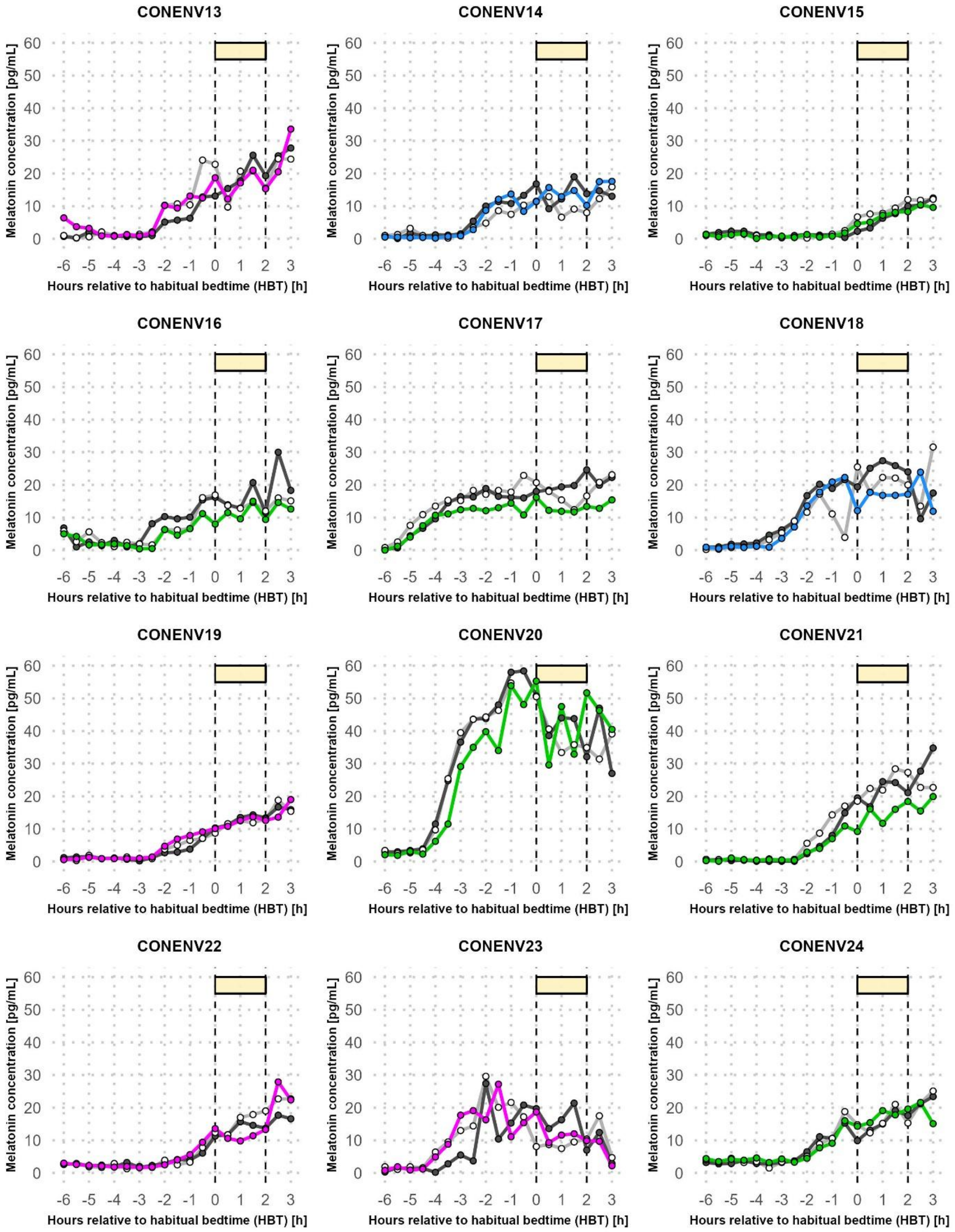

Figure S2. Individual salivary melatonin concentration profiles under each of the three light conditions for participants 13-24. Each panel represents the melatonin concentration (pg/ml) of a participant over time, relative to their habitual bedtime (HBT). Data are presented for the three experimental light conditions: Baseline Light (BL; black), Background Light (BG; gray) and Cone-Modulated Flickering Light (S, M-L, S+M+L; blue, green, pink). The dashed vertical lines indicate the duration of light exposure.

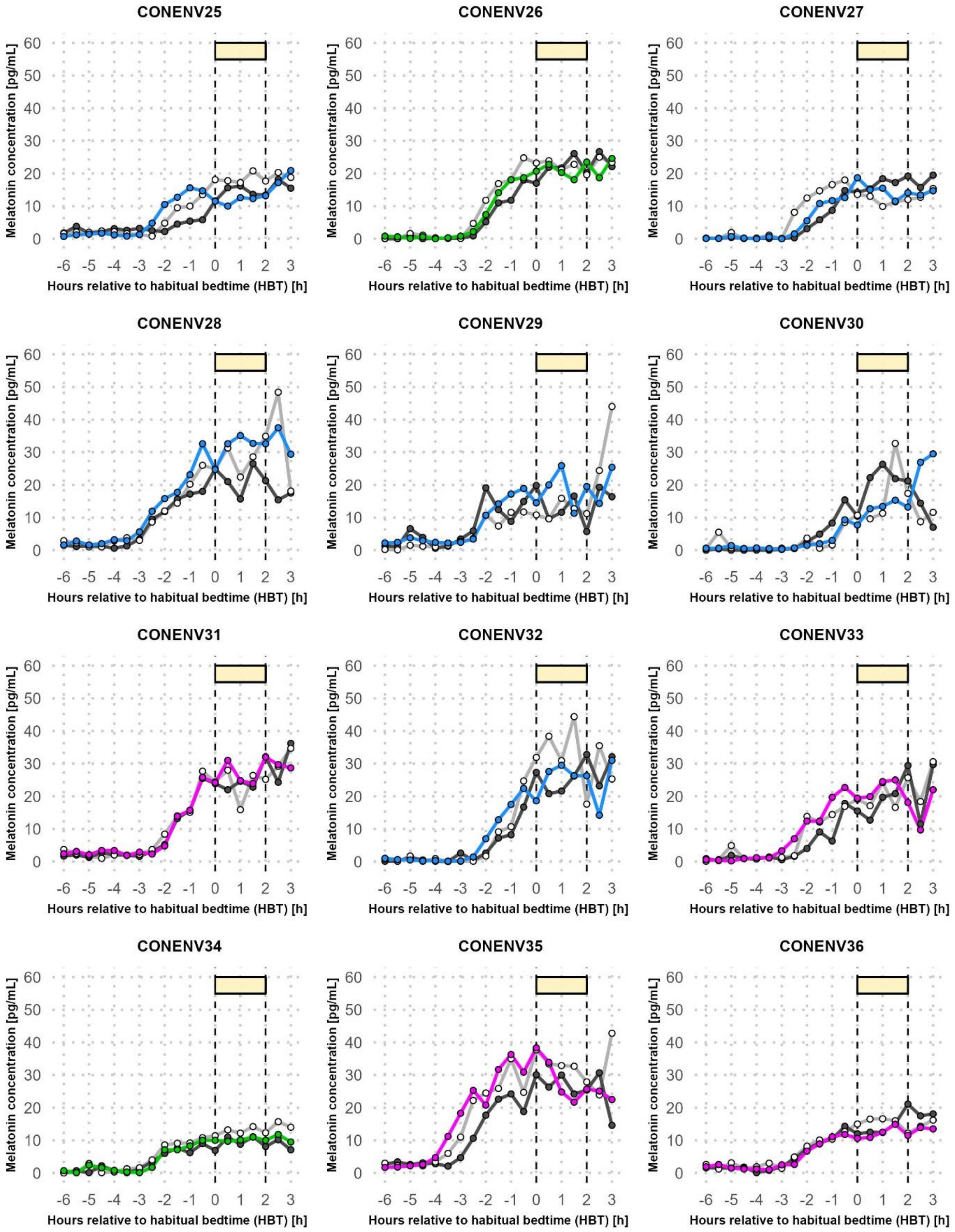

Figure S3. Individual salivary melatonin concentration profiles under each of the three light conditions for participants 25-36. Each panel represents the melatonin concentration (pg/ml) of a participant over time, relative to their habitual bedtime (HBT). Data are presented for the three experimental light conditions: Baseline Light (BL; black), Background Light (BG; gray) and Cone-Modulated Flickering Light (S, M-L, S+M+L; blue, green, pink). The dashed vertical lines indicate the duration of light exposure.

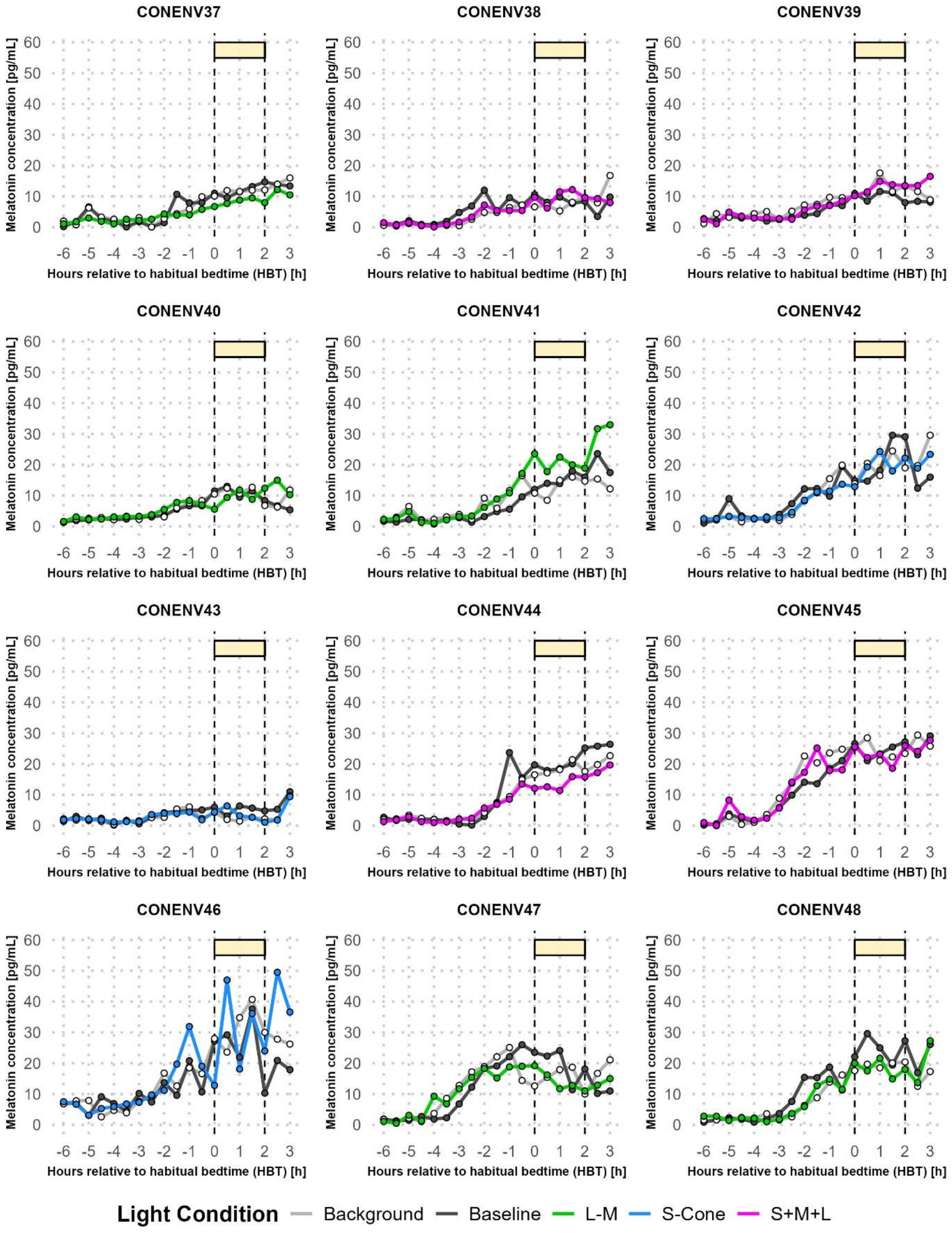

Figure S4. Individual salivary melatonin concentration profiles under each of the three light conditions for participants 37-48. Each panel represents the melatonin concentration (pg/ml) of a participant over time, relative to their habitual bedtime (HBT). Data are presented for the three experimental light conditions: Baseline Light (BL; black), Background Light (BG; gray) and Cone-Modulated Flickering Light (S, M-L, S+M+L; blue, green, pink). The dashed vertical lines indicate the duration of light exposure.

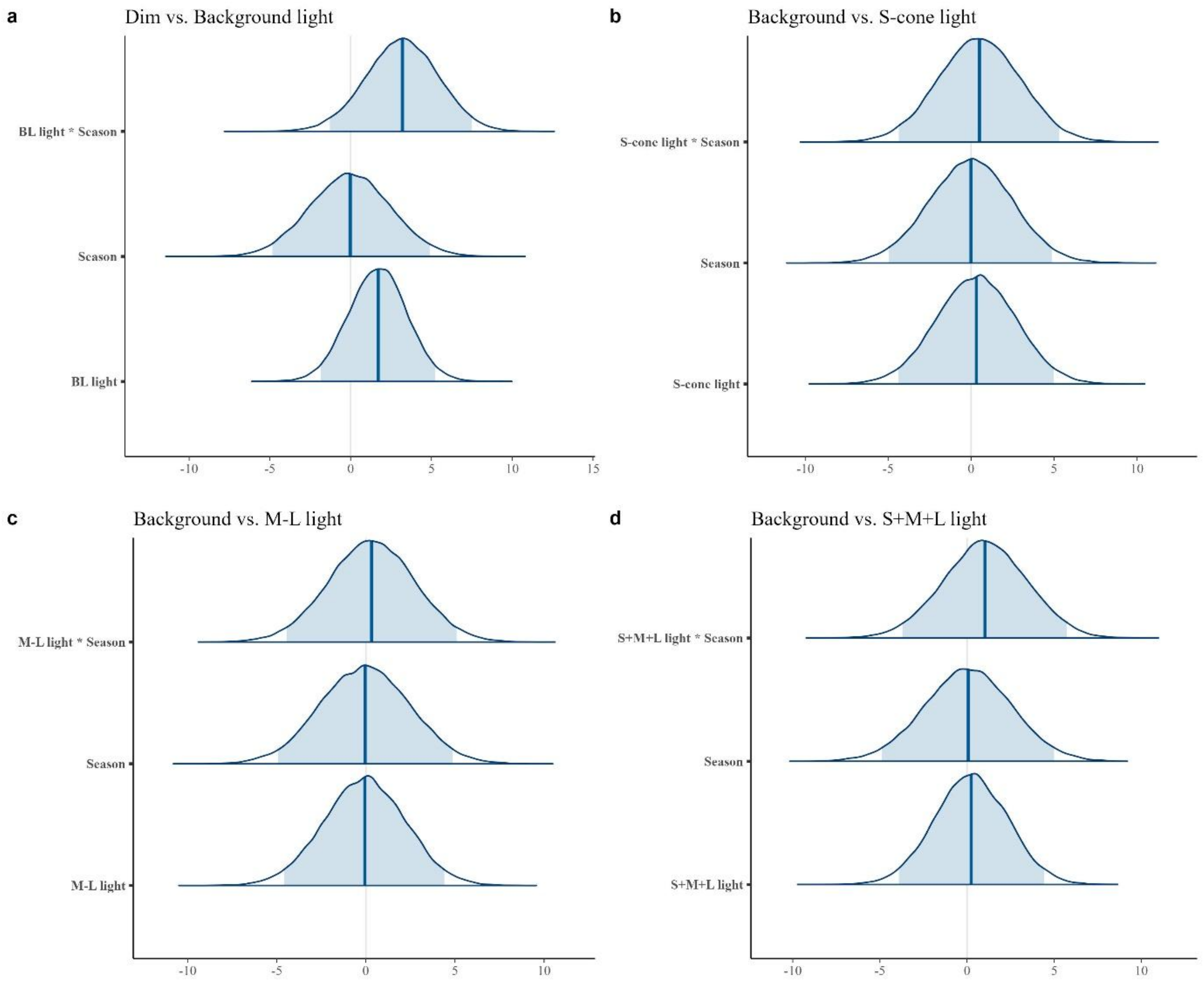

Figure S5. Posterior distributions of melatonin suppression (AUC) across light conditions. Posterior distribution plots for the estimated effects of (a) dim light vs. background light, (b) background light vs. S-cone flicker light, (c) background light vs. M-L flicker light, and (d) background light vs. S+M+L flicker light on melatonin area under the curve (AUC). Each panel shows the posterior distribution of effect estimates, with vertical lines representing the median of the posterior samples. The shaded regions indicate the 95% credible intervals.

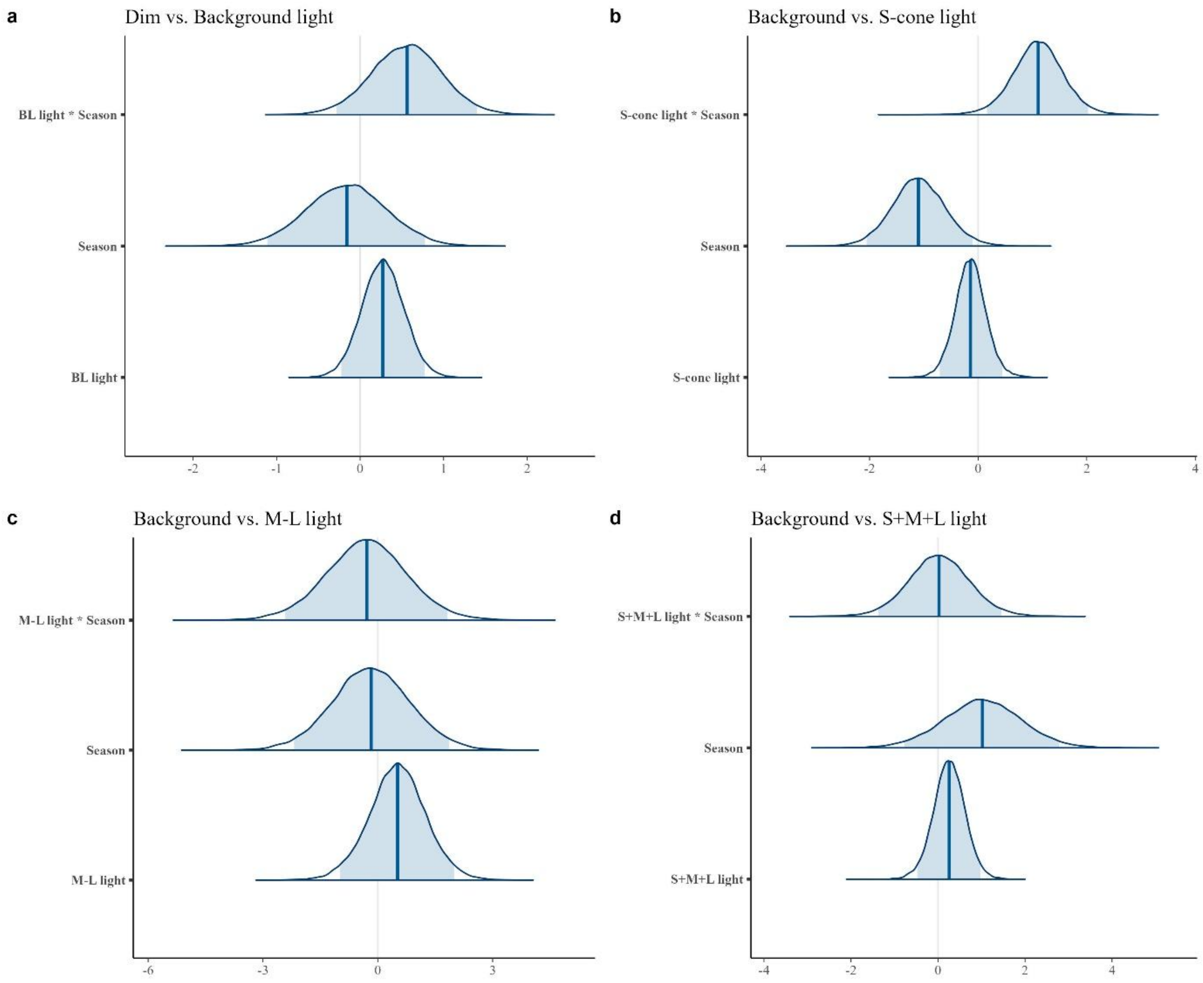

Figure S6. Posterior distributions of subjective alertness (KSS) across light conditions. Posterior distribution plots for the estimated effects of (a) dim light vs. background light, (b) background light vs. S-cone flicker light, (c) background light vs. M-L flicker light, and (d) background light vs. S+M+L flicker light on subjective alertness (KSS). Each panel shows the posterior distribution of effect estimates, with vertical lines representing the median of the posterior samples. The shaded regions indicate the 95% credible intervals.

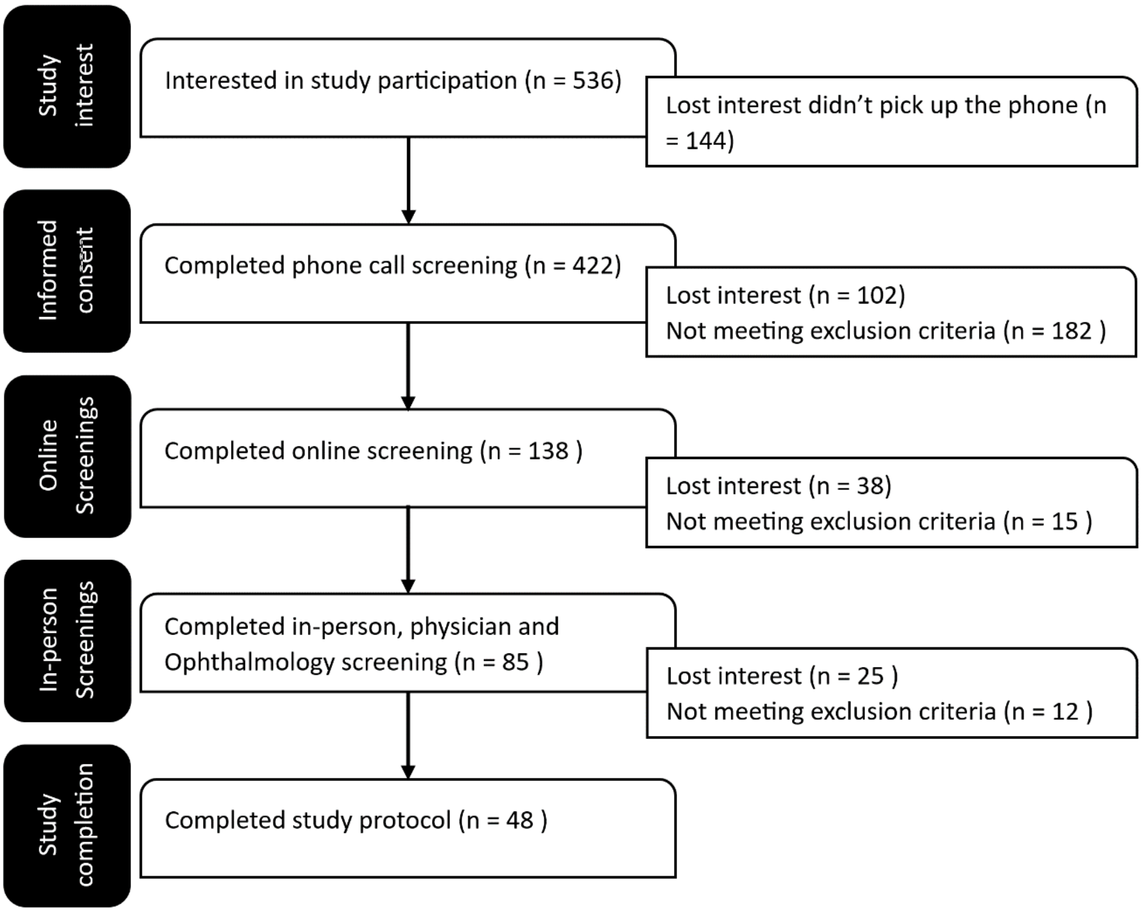

Figure S7. Study flowchart from initial interest to completion the study sessions. A total of 536 volunteers initially expressed interest in participating in the study, of whom 422 attended the mandatory telephone information session. Of these, 138 participants signed the informed consent form and completed the online screening. Of these, 85 attended the in-person, physician and ophthalmological screening. During the process, 25 participants lost interest, 7 were excluded due to non-compliance with the agreed sleep-wake schedule, 2 were excluded due to a positive drug test, and 3 withdrew after the first study session. In the end, 48 participants completed the study.

Table S1. The Bayes Factor (BF) interpretation based on Jeffreys (1961)

Bayes Factor	Interpretation
**> 100**	Decisive evidence for H1
**30 - 100**	Very strong evidence for H1
**10 - 30**	Strong evidence for H1
**3 - 10**	Moderate evidence for H1
**1 - 3**	Anecdotal evidence for H1
**1**	No evidence
**1/3 - 1**	Anecdotal evidence for H0
**1/10 – 1/3**	Moderate evidence for H0
**1/30 - 1/10**	Strong evidence for H0
**1/100 - 1/30**	Very strong evidence for H0
**< 1/100**	Decisive evidence for H0

Table S2. Individual melatonin suppression percentages across seasons (winter or summer).
Melatonin suppression was calculated as $((AUC_{BL} - AUC_{BG}) / AUC_{BL}) \times 100$ for each participant

Participant ID	Season	Melatonin Suppression
**CONE01**	Winter	33.40
**CONE02**	Winter	24.92
**CONE03**	Winter	-3.80
**CONE04**	Summer	7.73
**CONE05**	Winter	2.26
**CONE06**	Winter	37.95
**CONE07**	Winter	34.53
**CONE08**	Winter	9.92
**CONE09**	Winter	-3.64
**CONE10**	Winter	11.06
**CONE11**	Winter	13.75
**CONE12**	Winter	68.76
**CONE13**	Winter	6.07
**CONE14**	Winter	31.14
**CONE15**	Winter	-45.62
**CONE16**	Winter	9.45
**CONE17**	Winter	18.31
**CONE18**	Summer	15.13
**CONE19**	Summer	7.86
**CONE20**	Summer	9.14
**CONE21**	Summer	-11.42
**CONE22**	Summer	-14.31
**CONE23**	Summer	45.93
**CONE24**	Summer	-4.26
**CONE25**	Summer	-27.28
**CONE26**	Summer	-1.13
**CONE27**	Summer	30.47
**CONE28**	Summer	-30.01
**CONE29**	Summer	2.67

**CONE30**	Summer	21.51
**CONE31**	Summer	2.36
**CONE32**	Summer	-40.34
**CONE33**	Summer	-6.14
**CONE34**	Summer	-33.98
**CONE35**	Summer	-21.75
**CONE36**	Summer	-10.87
**CONE37**	Summer	1.16
**CONE38**	Summer	18.66
**CONE39**	Summer	-27.48
**CONE40**	Summer	-1.13
**CONE41**	Summer	12.77
**CONE42**	Summer	7.69
**CONE43**	Summer	54.12
**CONE44**	Summer	6.58
**CONE45**	Summer	0.51
**CONE46**	Summer	-19.21
**CONE47**	Summer	19.46
**CONE48**	Winter	21.68

Table S3. Intra-individual variability of melatonin suppression (AUC) and subjective alertness (KSS) across seasons. The standard deviation (SD) of individual melatonin area under the curve (AUC_intra_SD) and Karolinska Sleepiness Scale scores (KSS_intra_SD) for each participant are presented. Higher values indicate greater intra-individual variability in melatonin suppression or alertness ratings

Participant ID	Season	AUC_intra_SD	KSS_intra_SD
**CONE01**	Winter	25.64344	0
**CONE02**	Winter	16.88271	0.83887
**CONE03**	Winter	9.06371	0.509175
**CONE04**	Summer	2.241837	1.527525
**CONE05**	Winter	4.253234	1.154701
**CONE06**	Winter	7.420467	0.509175
**CONE07**	Winter	15.36563	0.3849
**CONE08**	Winter	18.53706	0.19245
**CONE09**	Winter	6.011725	1.333333
**CONE10**	Winter	6.399284	1.170628
**CONE11**	Winter	8.38436	0.333333
**CONE12**	Winter	56.57515	0.83887
**CONE13**	Winter	3.824591	0.881917
**CONE14**	Winter	9.613315	1.170628
**CONE15**	Winter	5.467175	1.575272
**CONE16**	Winter	8.39747	0.509175
**CONE17**	Winter	14.15106	2.0367
**CONE18**	Summer	17.13702	0.19245
**CONE19**	Summer	2.013289	0.509175

**CONE20**	Summer	7.911437	1.26198
**CONE21**	Summer	19.72849	1.01835
**CONE22**	Summer	8.53527	0.3849
**CONE23**	Summer	14.92568	0.57735
**CONE24**	Summer	4.24539	0.333333
**CONE25**	Summer	13.3083	0.19245
**CONE26**	Summer	3.21455	0.666667
**CONE27**	Summer	10.31956	1.01835
**CONE28**	Summer	21.53406	0.3849
**CONE29**	Summer	14.06026	1.01835
**CONE30**	Summer	17.19334	0.509175
**CONE31**	Summer	6.766831	0.333333
**CONE32**	Summer	21.20786	0
**CONE33**	Summer	6.317898	1.154701
**CONE34**	Summer	6.946042	3.371998
**CONE35**	Summer	12.57103	0.96225
**CONE36**	Summer	6.681878	0.19245
**CONE37**	Summer	7.813503	0.333333
**CONE38**	Summer	5.533308	1.20185
**CONE39**	Summer	6.495383	0.693889
**CONE40**	Summer	3.012612	0.7698
**CONE41**	Summer	15.19723	0.333333
**CONE42**	Summer	3.458805	0.509175
**CONE43**	Summer	5.575019	0.333333
**CONE44**	Summer	13.24802	0.83887
**CONE45**	Summer	3.991554	1.575272
**CONE46**	Summer	10.37862	0.19245
**CONE47**	Summer	11.62024	0.666667
**CONE48**	Winter	13.76554	3.40479

Table S4. The Inter-individual variability in melatonin suppression (AUC) and subjective alertness (KSS) across seasons. The mean, standard deviation (SD) and coefficient of variation (CV) for melatonin area under the curve (AUC) and Karolinska Sleepiness Scale (KSS) scores in winter (n = 17) and summer (n = 31) are presented. The CV is the ratio of the standard deviation to the mean and provides a normalised measure of the variability between individuals within each season.

**Season**		**Melatonin AUC**	**KSS**
**Winter (n = 17)**	Mean	74.17	6.95
	SD	31.71	1.60
	CV	0.42	0.23
**Summer (n = 31)**	Mean	71.29	6.85
	SD	31.76	1.67
	CV	0.44	0.24

Table S5. The Inter-individual variability in Karolinska Sleepiness Scale (KSS) scores across light conditions. Mean, standard deviation (SD) and coefficient of variation (CV) for all participants (N = 48) and for three subgroups (n = 16 each) under different light conditions: Baseline light (BL), Background light (BG), S-cone (S), M-L (ML) and S+M+L (SML) are presented.

	Condition	KSS_mean	KSS_SD	KSS_CV
**All participants (n = 48)**	BG	6.625	1.737672	0.26229
	BL	7.097222	1.456961	0.205286
**Group 1 (n = 16)**	BG	7.333333	1.148268	0.156582
	S	7.604167	0.800174	0.105228
**Group 2 (n = 16)**	BG	5.9375	2.164422	0.364534
	ML	6.354167	2.172109	0.34184
**Group 3 (n = 16)**	BG	6.604167	1.55501	0.235459
	SML	6.875	1.780033	0.258914

Table S6. Participants' characteristics in different cone-modulated flickering groups. Data are expressed as mean (± SD). BMI: Body Mass Index; BDI-II: Beck Depression Inventory; μMCTQ: Ultra-Short Version of the Munich ChronoType Questionnaire; PSQI: Pittsburgh Sleep Questionnaire; ESS: Epworth Sleepiness Scale; Visual Acuity with single Landolt C; Farnsworth Munsell 100 Hue test; Cambridge Colour Test Trivector; Protan, Deutan, and Tritan

Descriptives		Group 1 (S-Cone)	Group 2 (M-L)	Group 3 (S+M+L)
**Age**		25.72 (3.74)	26.11 (5.08)	23.50 (4.17)
**BMI**		22.10 (2.44)	22.31 (2.87)	22.93 (1.61)
**BDI_II**		2.38 (2.60)	2.13 (2.55)	4.63 (4.75)
**μMCTQ**		4.38 (0.70)	3.98 (0.83)	3.95 (0.91)
**PSQI**		3.31 (1.20)	2.88 (1.75)	3.44 (1.36)
**ESS**		6.31 (3.32)	5.25 (2.86)	5.63 (2.73)
**Visual Acuity**		1.96 (0.44)	1.78 (0.42)	1.99 (0.32)
**Farnsworth**		12.38 (3.88)	13.38 (4.43)	13.38 (5.45)
**CCT**	**Protan**	4.73 (1.79)	5.13 (1.56)	4.08 (1.37)
	**Deutan**	4.77 (1.61)	4.66 (1.55)	4.57 (1.69)
	**Tritan**	6.70 (2.79)	6.23 (2.30)	6.24 (2.64)

Table S7. Inclusion and exclusion criteria of online, in-person, physician and ophthalmology, and every session screening

	Aspect	Assessment modality	Exclusion criterion and cut-off
**Online screening**	Age	Self-report	<18 years and >35 years
	BMI	Self-reported height and weight	<18.5 and >29.9
	Pregnancy (only female)	Self-report	'Yes' response
	Use of hormonal contraceptives (only female)	Self-report	'Yes' response
	Lactation or breastfeeding (only female)	Self-report	'Yes' response

	Menstrual cycle (only female)	Reproductive Status Questionnaire for Menstrual Cycle Studies (ref)	
	Color vision deficiency (only for category 2)	Ishihara Test (r	' Yes ' response
	Chronotype	Ultra-short Munich Chronotype Questionnaire (µMCTQ)	≤ 2 and ≥7
	Sleep duration	Ultra-short Munich Chronotype Questionnaire (µMCTQ)	< 6 and > 9
	Sleep quality	Pittsburgh Sleep Quality Index, PSQI	>5
	Smoking	Self-report	>0
	Substance abuse	Alcohol Use Disorders Identification Test, AUDIT	>7
	Depressive symptoms	BDI-II	>13
	High myopia	Self-report from prescription information	< -6 diopters
	High hyperopia	Self-report from prescription information	> +6 diopters
	Transmeridian travel (>2 zones) <1 month prior to first session	Self-report	'Yes' response
	Shift work <3 months prior to study	Self-report	'Yes' response
	Current participation in other clinical trials	Self-report	'Yes' response
	Any ophthalmological or optometric conditions (cataract, glaucoma, retinal detachment, macular conditions, chronic inflammations, eye injuries or operations)	Self-report	Any 'Yes' response
	Any general health concerns or disorders, including heart and cardiovascular, neurological, nephrological, endocrinological and psychiatric conditions	Self-report	Any 'Yes' response
	Any chronic medication affect on sleep	Self-report	Any 'Yes' response
**in-person screening**	BMI	Measured height and weight	<18.5 and >29.9
	Pregnancy test (only women)	M-Budget pregnancy test	Positive test
	Normal color vision	Cambridge Color Test, Farnsworth Munsell 100 Hue Test	Protan >10, Detran > 10, Tritan > 15, and 100 Hue score > 40
	Normal best-corrected visual acuity (BCVA)	Landolt C test	Visus < 0.5
	Ability to understand study language	In-person interaction with the experimenter	Experimenter judgment
**Physician and ophthalmology screening**	Colour vision deficiency	Check by ophthalmologist	Ophthalmologist judgment
	Risk of angle-closure glaucoma	Check by ophthalmologist	Ophthalmologist judgment
	Any ophthalmological or optometric conditions (cataract, glaucoma, retinal detachment, macular conditions, chronic inflammations, eye injuries or operations)	Check by ophthalmologist	Ophthalmologist judgment

	Any general health concerns or disorders, including heart and cardiovascular, neurological, nephrological, endocrinological and psychiatric conditions	Check by physician	Physician judgment
	Any chronic medication affects on sleep	Check by physician	Physician judgment
**Every session screening**	Drug use (AMP, BZD, COC, MOR/OPI, MTD and THC)	Drug-Screen-Multi 6; nal von Minden	Any positive test
	Alcohol use	Breathalyzer ACE X	>0.05
	Sleep-wake times in 5 days prior to each experimental session	Actigraphy record and sleep diary	>1 deviation from ±30 minute window sleep and wake-up time
	Ability to follow study instructions	In-person interaction with the experimenter	Experimenter judgment
